# James W. White, A.M., M.D., D.D.S.

**Published:** 1891-07

**Authors:** 


### JAMES W. WHITE, A.M., M.D., D.D.S.

As we were about going to press with the last number we, in common with others, were shocked by the sudden death of Dr. White. His long career of activity as editor of the *Dental Cosmos* and his connection with the S. S. White Dental Manufacturing Company as its president has made his name and work familiar to thousands who never knew him personally. The death of his brother, Dr. S. S. White, threw a heavy responsibility upon him in the reorganization of that house, and its subsequent great success must, in a large degree, be attributed to his energy and skill as an organizer. He was a man capable of assuming heavy responsibilities, and the burden seemed to affect him less than most similarly situated.

While in no sense a practical dentist, so intimate were the relations between the profession and himself that few realized the fact, and he was ever a welcome visitor at the various conventions he was in the habit of attending.

In his difficult position as editor of the *Cosmos* he necessarily created antagonisms; but those who knew him best always found him genial and ever ready to do a kindly act. He lived in his work, and that was to broaden life and to make those with whom he was brought in contact better for his influence.

His charity was not of the obtrusive kind, but was a constant factor in his life. It was a pleasure to him to give of his medical knowledge gratuitously, and who can number the blessings he showered in many silent ways on those who needed help?

He was no idealist, and had but little sympathy for the sentimental side of life. As an editor he was a severe and uncompromising critic, which led many to erroneous opinions as to his real character.

The grave has closed over an earnest man, true in his social relations, progressive in all that led to the best growth of his native city, and faithful to all that goes to make for the highest good of all peoples.

